# Contrast-dependent orientation discrimination in the mouse

**DOI:** 10.1038/srep15830

**Published:** 2015-10-29

**Authors:** Minghai Long, Weiqian Jiang, Dechen Liu, Haishan Yao

**Affiliations:** 1 Institute of Neuroscience and State Key Laboratory of Neuroscience, Shanghai Institutes for Biological Sciences, Chinese Academy of Sciences, Shanghai 200031, China; 2 University of Chinese Academy of Sciences, Shanghai 200031, China

## Abstract

As an important animal model to study the relationship between behaviour and neural activity, the mouse is able to perform a variety of visual tasks, such as orientation discrimination and contrast detection. However, it is not clear how stimulus contrast influences the performance of orientation discrimination in mice. In this study, we used two task designs, two-alternative forced choice (2AFC) and go/no-go, to examine the performance of mice to discriminate two orthogonal orientations at different contrasts. We found that the performance tended to increase with contrast, and the performance at high contrast was better when the stimulus set contained a single contrast than multiple contrasts. Physiological experiments in V1 showed that neural discriminability of two orthogonal orientations increased with contrast. Furthermore, orientation discriminability of V1 neurons at high contrast was higher in the single than in the multiple contrast condition, largely due to smaller response variance in the single contrast condition. Thus, the performance of mice to discriminate orientations at high contrast is adapted to the contrast range in the stimuli, partly attributed to the contrast-range dependent capacity of V1 neurons to discriminate orientations.

The responses of visual cortical neurons are modulated by stimulus contrast. A typical contrast response function exhibits monotonic increase in response within a limited range of low contrasts, and compression and saturation at high contrast range^1^,^2^,^3^. It has been shown that a variety of stimulus selectivities in visual cortex depend on stimulus contrast. For example, direction selectivity changed with stimulus contrast for some neurons in cat areas 17 and 18^4^,^5^,^6^. Spatial summation and spatial frequency tuning were contrast-dependent in V1^7^,^8^,^9^,^10^. The structure of orientation tuning curves varied with contrast for V1 neurons in ferret and mouse and color-responsive V1 neurons in monkey^11^,^12^,^13^. At perceptual level, stimulus contrast affects the judgments of human subjects on many visual tasks, including motion discrimination^14^,^15^, perceived speed of moving targets^16^,^17^, perceived spatial frequency^18^,^19^, orientation and spatial frequency discrimination^20^,^21^, and the influence of contextual stimuli on orientation discrimination^22^. Thus, both physiological and psychophysical studies demonstrated that cortical processing of stimulus features are dependent on stimulus contrast.

The mouse has emerged to be an important animal model to examine the relationship between neural activity and behaviour^23^. Although V1 neurons in mouse have low spatial resolution, they are highly selective to stimulus features such as orientation, spatial frequency, temporal frequency, and contrast^3^,^24^,^25^,^26^,^27^. A variety of behavioural methods and task designs have been developed to probe mouse vision. Mice can be trained to perform tasks of motion discrimination^28^, orientation discrimination^29^,^30^,^31^,^32^, detection of orientation and contrast changes^33^, and contrast detection^34^,^35^,^36^. Recent studies revealed that contrast has dramatic effects on the response amplitudes and orientation tunings in mouse V1^3^,^12^; however, few studies examined how stimulus contrast influences the behavioural performance of orientation discrimination in mice and whether the performance of orientation discrimination is adapted to the contrast range in the stimuli. Furthermore, as different task designs may exhibit different degrees of vulnerability to changes in the animal’s decision criterion^37^, it is also important to compare behavioural results between different task designs^38^.

In this study, we have examined how stimulus contrast influences the performance of orientation discrimination in mice using a 2AFC^34^ and a go/no-go^30^ task. We found that the behavioural performance increased with contrast, and the effect was more evident for the 2AFC task. When the stimulus set contained a single high contrast, the performance was better than that at the same high contrast in the multiple contrast condition. By measuring the contrast response functions for preferred and orthogonal orientations in V1, we showed that neural discriminability at high contrast was better in the single than in the multiple contrast condition. Thus, the performance of mice to discriminate two high-contrast orientations is adapted to the contrast range in the stimuli. Such effect may be partly mediated by the dependence of orientation discriminability on the contrast range in V1.

## Results

### Performance of mouse orientation discrimination at different contrasts

**Figure 1:**
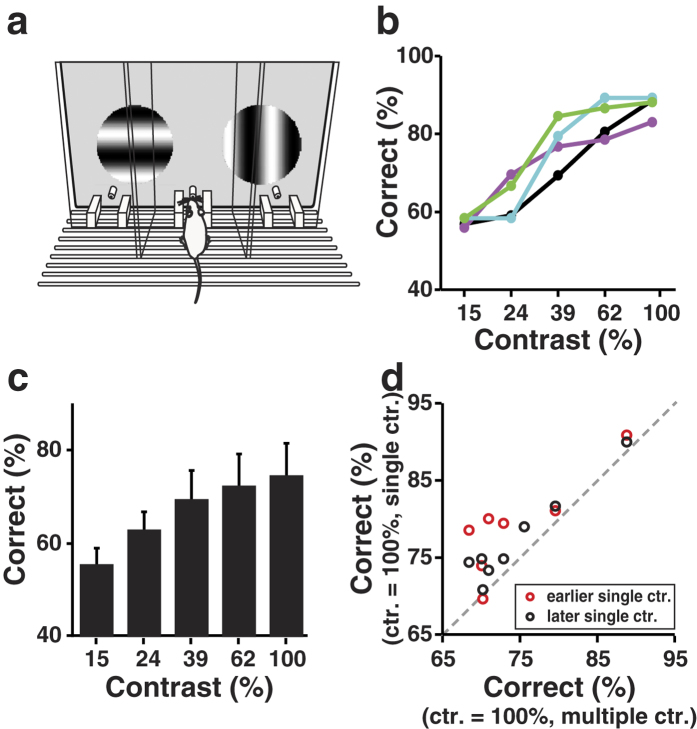
Dependence of orientation discrimination performance on contrast in the 2AFC task. (**a**) Schematic illustration of the behavioural apparatus. (**b**) Performance in the multiple contrast condition for an example mouse. Each color represents one session. (**c**) Correct rate at each contrast in the multiple contrast condition. Spearman’s rank correlation coefficient *r* = 1, one-tailed *P* = 0.008; *P* = 2.4 × 10^−7^ for ANOVA. Error bars, s.d., *n* = 8 mice. (**d**) Comparison of performance at 100% contrast between the single contrast condition and the multiple contrast condition. Dashed line, the diagonal line. Red (black) circles represent the earlier (later) single contrast condition tested before (after) the multiple contrast condition. *P* = 0.008 for red data points, *P* = 0.004 for black data points, one-tail, Wilcoxon signed rank test.

We used a 2AFC and a go/no-go task design to examine the performance of mouse to discriminate two orthogonal orientations at different contrasts (Methods). For the 2AFC task, freely moving mice were trained in a behaviour chamber, which contained three nose-poking ports on a wall facing an LCD monitor^34^ (Fig. 1a). Nose poking in the center port would trigger the presentation of two visual stimuli, target and non-target, each on one side of the monitor. The target stimulus was a static grating at vertical orientation and the non-target was one at horizontal orientation. The mouse indicated the target stimulus by poking its nose into the port corresponding to the side where the target was presented. Choosing the correct port was rewarded by water, and choosing the incorrect port was followed by a timeout period.

The training of 2AFC task consisted of five steps (Methods). In an early step, only the target stimulus was presented on the left or right side of the monitor, and the mice were required to choose the port corresponding to the side the target was presented. For this step, mice learned to choose the correct port within 22 ± 4.1 (mean ± s.d.) sessions, during which the correct rate increased from 51.9% ± 4.1% to 82.4% ± 3.9% (mean ± s.d., *n* = 8, *P* = 0.004, one-tail, Wilcoxon signed rank test). In the next step, both target and non-target stimuli were presented on the monitor, and the stimuli were at a single contrast of 100%. For this step, choosing the port at the side the target was presented was counted as a correct trial, and the correct rate of mice could reach 79.1 ± 6.1% (mean ± s.d.) within 3 sessions. We then tested the mice’s performance in the multiple contrast condition, in which the stimulus set contained five contrasts ranging from 15% to 100%. Each block included four trials of the same contrast, and the contrasts in different blocks were randomized. Figure 1b shows the performance of an example mouse, whose correct rate increased with contrast in all sessions. For all mice tested, the correct rate clearly increased with contrast (*n* = 8, Spearman’s rank correlation coefficient *r* = 1, one-tailed *P* = 0.008; and *P* = 2.4 × 10^−7^ for ANOVA, Fig. 1c), indicating that the mice’s performance of orientation discrimination improved with contrast. After measuring the mice’s performance with multiple contrasts, we re-measured their performance at a single contrast of 100%. We found that the performance at 100% contrast in the single contrast condition was significantly better than that in the multiple contrast condition (*P* < 0.01, one-tail, Wilcoxon signed rank test, Fig. 1d). The correct rates at 100% contrast in the single contrast condition measured before and after the multiple contrast condition were both significantly larger than that in the multiple contrast condition (*P* = 0.008 for the earlier single contrast condition, *P* = 0.004 for the later single contrast condition, one-tail, Wilcoxon signed rank test, Fig. 1d), suggesting that the worse performance at high contrast in the multiple contrast condition could not be explained by the length of training. Thus, the performance of orientation discrimination at high contrast depended on the range of contrast in the stimuli.

**Figure 2:**
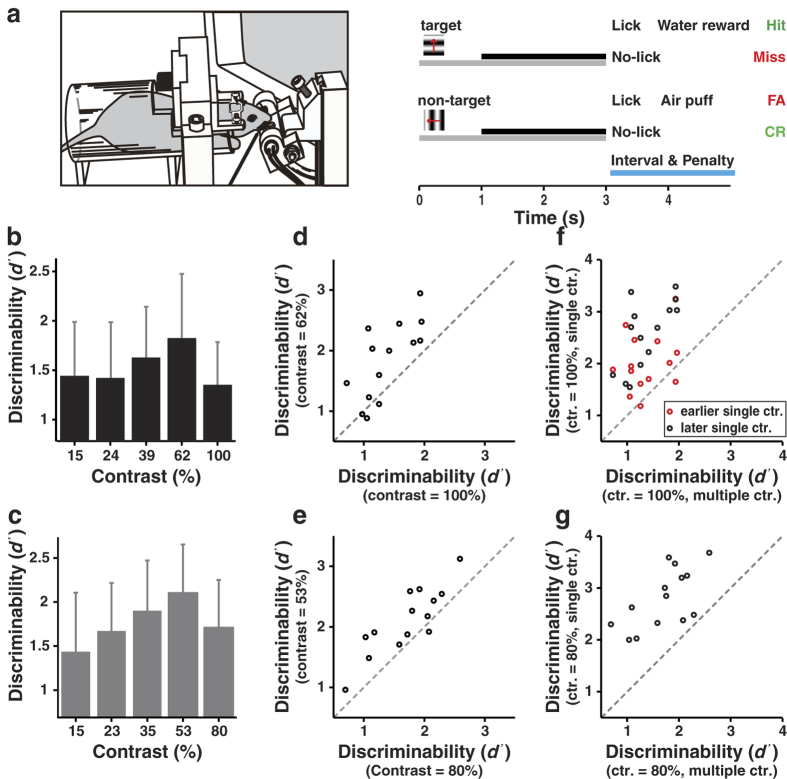
Dependence of orientation discrimination performance on contrast in the go/no-go task. (**a**) Left, schematic illustration of the behavioural apparatus. Right, The timing of trial events and behavioural outcomes. Gray bar from 0 to 3 s indicates the duration of visual stimuli, black bar indicates the response window, and blue bar indicates the inter-stimulus interval. FA, false alarm. CR, correct rejection. (**b**) Discriminability in the multiple contrast condition in which the contrasts ranged from 15% to 100% (*P* = 0.15, ANOVA). (**c**) Discriminability in the multiple contrast condition in which the contrasts ranged from 15% to 80% (*P* < 0.05, ANOVA). Error bars, s.d., *n* = 14 mice. (**d**) Comparison of discriminability between 62% and 100% contrasts for the multiple contrast condition in which the contrasts ranged from 15% to 100%. Dashed line, the diagonal line. *P* = 0.001, one-tail, Wilcoxon signed rank test. (**e**) Comparison of discriminability between 53% and 80% contrasts for the multiple contrast condition in which the contrasts ranged from 15% to 80%. *P* = 4.3 × 10^−4^, one-tail, Wilcoxon signed rank test. (**f**) Comparison of discriminability at 100% contrast between the single contrast condition and the multiple contrast condition. Red (black) circles represent the earlier (later) single contrast condition tested before (after) the multiple contrast condition. *P* = 6.1 × 10^−4^ for red data points, *P* = 6.1 × 10^−5^ for black data points, one-tail, Wilcoxon signed rank test. (**g**) Comparison of discriminability at 80% contrast between the single contrast condition and the multiple contrast condition. The single contrast condition was measured after the multiple contrast condition. *P* = 6.1 × 10^−5^, one-tail, Wilcoxon signed rank test.

We also used a go/no-go task^30^ to examine the orientation discrimination performance for a different group of mice. Head-fixed mice were trained to discriminate between a horizontally oriented grating drifting upward (target) and a vertically oriented grating drifting leftward (non-target) presented to the animal’s left eye (Fig. 2a, Methods). The mice indicated the stimulus orientation by licking or withhold licking during the stimulus presentation. During the response window of target presentation, licking was counted as a hit and followed by water reward, whereas no lick was counted as a miss. During the response window of non-target presentation, no lick was counted as a correct rejection, while licking was counted as a false alarm (FA) that was followed by a punishment of air puff to the facial skin. To quantify the behavioural discriminability of target from non-target, we computed a measure of *d*′ using the hit rate (fraction of trials with lick during target presentation) and FA rate (fraction of trials with lick during non-target presentation)^39^. Higher *d*′ values indicate better performance in orientation discrimination.

For the go/no-go task, we first measured the mice’s performance using stimuli at a single contrast of 100%. It took 13.1 ± 5.6 (mean ± s.d., *n* = 14) sessions for the performance to reach a threshold of *d*′ = 1^30^,^31^, and *d*′ significantly increased to 2.0 ± 0.6 (mean ± s.d.) after training (*n* = 14, *P* = 6.1 × 10^−5^, one-tail, Wilcoxon signed rank test, Supplementary Fig. S1). Training also decreased the FA rate and miss rate, and increased the mice’s lick efficiency^40^ (the ratio between the number of licks within the response window and the total number of licks in a target trial) (Supplementary Fig. S1), indicating that the mice understood the task structure.

After the mice learned the go/no-go task using stimuli at a single contrast of 100%, we trained them to perform the task in the multiple contrast condition, in which the stimulus set contained five contrasts ranging from 15% to 100% or from 15% to 80%. Overall, the *d*′ tended to increase with contrast, especially for the first four levels of contrast (Fig. 2b,c). For the go/no-go task, the performance at the highest contrast was worse than that at the second highest contrast (*P* = 0.001 between *d*′ at 100% and 62% contrasts, Fig. 2d; *P* = 4.3 × 10^−4^ between *d*′ at 80% and 53% contrasts, Fig. 2e; one-tail, Wilcoxon signed rank test). For the 2AFC task, in contrast, the performance at 100% contrast was significantly better than that at 62% contrast (*P* = 0.02, one-tail, Wilcoxon signed rank test). Thus, when multiple contrasts were interleaved in the stimuli in a go/no-go task, mice’s performance at the highest contrast tended to decrease.

For the go/no-go task, the *d*′ at high contrast (100% or 80%) was significantly higher in the single than in the multiple contrast condition (*P* < 5 × 10^−3^ at 100% contrast, *P* = 6.1 × 10^−5^ at 80% contrast, one-tail, Wilcoxon signed rank test, Fig. 2f,g). Thus, for both task designs, the performance at high contrast was dependent on the range of contrast in the stimuli.

**Figure 3:**
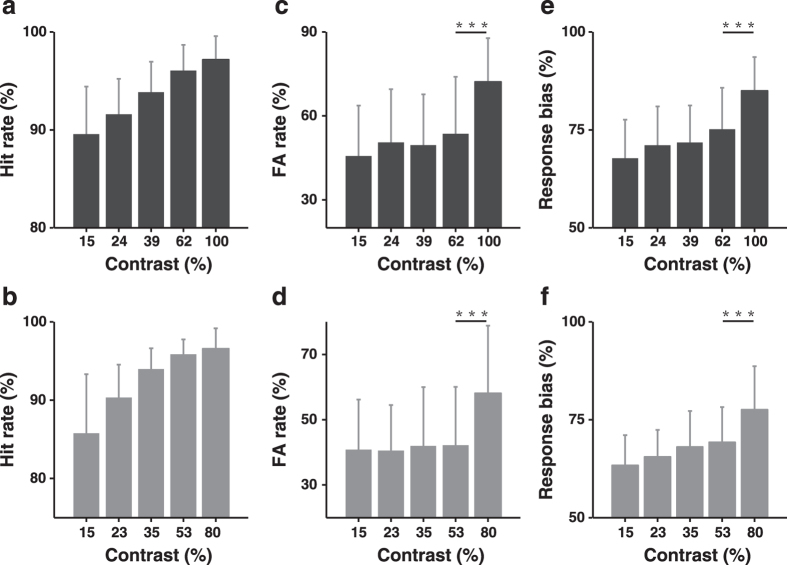
Hit rate, FA rate, and response bias in the multiple contrast condition during the go/no-go task. (**a**,**b**) Hit rate increased with contrast. Spearman’s rank correlation coefficient *r* = 1, *P* = 0.008. Upper panel, the contrasts ranged from 15% to 100%; lower panel, the contrasts ranged from 15% to 80%. (**c**,**d**) FA rate increased with stimulus contrast (*P* < 0.05, ANOVA). (**e**,**f**) Response bias increased with stimulus contrast. Spearman’s rank correlation coefficient *r* = 1, *P* = 0.008. Error bars, s.d., *n* = 14 mice. ***P < 0.001, Wilcoxon signed rank test.

It has been shown that go/no-go tasks are highly vulnerable to changes in the animal’s willingness to respond^37^. We thus examined whether the worse performance at the highest contrast in the multiple contrast condition during the go/no-go task is due to a change in response bias. For the mice tested with go/no-go task, the hit rate increased with stimulus contrast (Spearman’s rank correlation coefficient *r* = 1, *P* = 0.008, one-tail, Fig. 3a,b), and the FA rate rose prominently only when stimulus contrast reached the highest value of 100% or 80% (*P* = 6.1 × 10^−5^ between 62% and 100% contrasts, *P* = 6.1 × 10^−5^ between 53% and 80% contrasts, one-tail, Wilcoxon signed rank test, Fig. 3c,d). As a result, the response bias (the number of hits and FAs divided by the total number of stimuli) also increased from the second highest to the highest contrast (*P* < 2 × 10^−4^, one-tail, Wilcoxon signed rank test, Fig. 3e,f). While the hit rate at high contrast was above 90% in both the multiple and the single contrast conditions, the FA rate and response bias at high contrast increased substantially in the multiple contrast condition as compared with the single contrast condition (Supplementary Fig. S2). Thus, the worse performance at the highest contrast in the multiple contrast condition during the go/no-go task was largely due to the increase in response bias.

### Contrast-dependent orientation discrimination in V1

To examine whether the mice’s performance of orientation discrimination at high contrast can be explained by the response properties in V1, we measured the responses of V1 neurons using drifting grating stimuli at different orientations and contrasts (Methods). Similar to the behavioural experiments, the physiological experiments included stimulation conditions of multiple contrasts (ranging from 15% to 100%, or from 15% to 80%) and a single contrast (100% or 80%).

**Figure 4:**
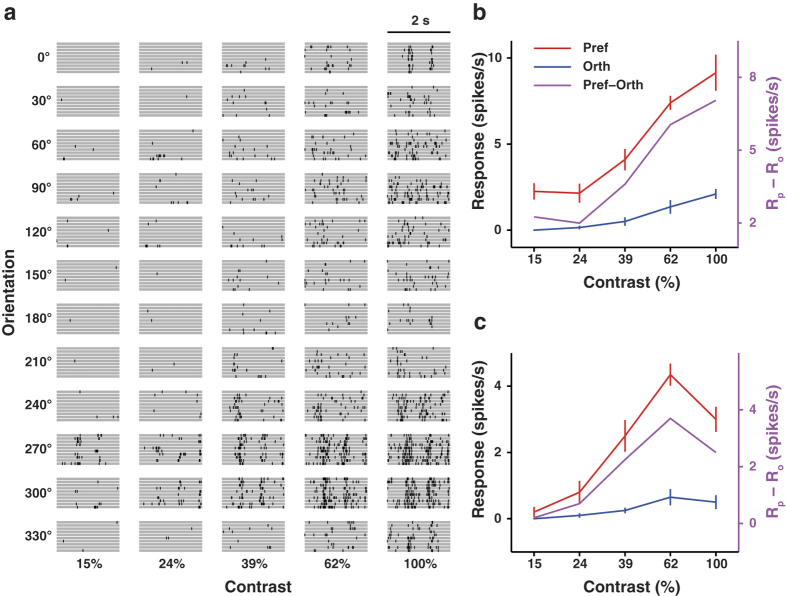
Responses of example V1 neurons to oriented gratings at different contrasts. (**a**) Spike rasters of an example neuron in response to drifting gratings at different orientations and contrasts. (**b**) Responses to preferred and orthogonal orientations for the cell in (**a**). Red, contrast response function for preferred orientation. Blue, contrast response function for orthogonal orientation. Magenta, response difference between preferred and orthogonal orientations as a function of contrast. (**c**) Same as described in (**b**) for another example cell. Error bars, s.e.m.

**Figure 5:**
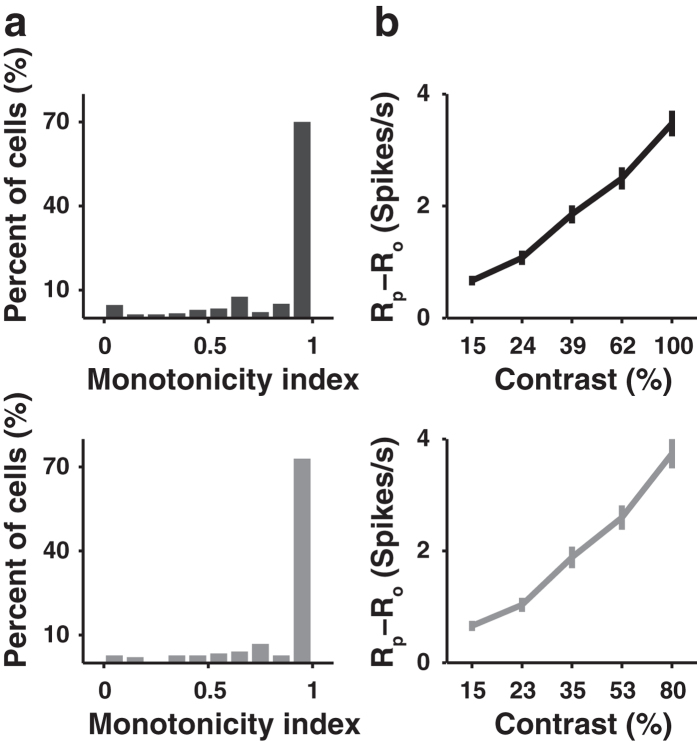
Dependence of P-O response difference on contrast for V1 neurons. (**a**) Upper: distribution of monotonicity index for the responses measured with contrasts ranging from 15% to 100% (*n* = 237, including 188 neurons from anaesthetized mice and 49 neurons from awake mice). Lower: distribution of monotonicity index for the responses measured with contrasts ranging from 15% to 80% (*n* = 148 neurons from anaesthetized mice). (**b**) Response difference between preferred and orthogonal orientations averaged over all neurons. Upper: contrast range of 15% to 100%, Spearman’s rank correlation coefficient *r* = 1, *P* = 0.008, *n* = 237. Lower: contrast range of 15% to 80%, Spearman’s rank correlation coefficient *r* = 1, *P* = 0.008, *n* = 148. Error bars, s.e.m.

Figure 4a shows the spike rasters of an example neuron in response to grating stimuli at different orientations and contrasts. For each contrast, we compared the responses to the preferred and orthogonal orientations (orthogonal orientation = preferred orientation + 90^°^) by computing a P-O difference defined as R_p_ - R_o_, where R_p_ and R_o_ represent the responses to preferred and orthogonal orientations, respectively (Fig. 4b,c, Methods). With the increase in contrast, the P-O difference exhibited a monotonic increasing profile for some and a non-monotonic profile (i.e., the maximal P-O difference is not at the highest contrast) for other neurons. By analogy to previous studies in cat and monkey visual cortex^5^,^41^, for each neuron we quantified the dependence of P-O difference on contrast using a monotonicity index (MI, Methods). A curve with MI = 1 was defined as monotonic with contrast and MI < 1 as non-monotonic^5^. We found that MI was 1 for the majority of neurons (Fig. 5a) and the P-O difference averaged over the population increased monotonically with contrast (*n* = 237 and 148 for the two types of multiple contrast conditions, Fig. 5b). Thus, the response difference between preferred and orthogonal orientations increased with stimulus contrast in V1.

**Figure 6:**
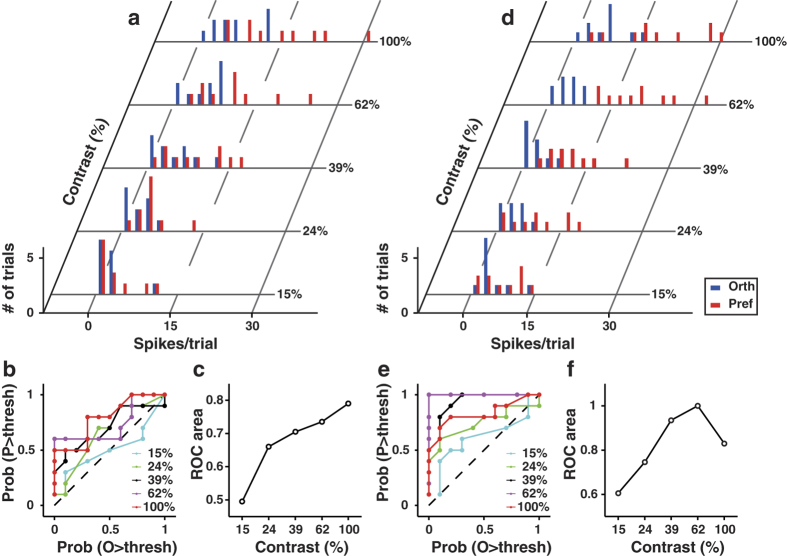
ROC analysis and neurometric function. (**a**) Distribution of responses from an example neuron in response to preferred (red bars) and orthogonal (blue bars) orientations at five contrast levels. (**b**) Receiver operating characteristic (ROC) curves for the responses to the five contrasts shown in (**a**). For each ROC curve, the probability that the response to the preferred orientation exceeds a given criterion value is plotted against that to the orthogonal orientation. (**c**) Neurometric function for the responses shown in (**a**). The ROC area for each contrast was computed from the corresponding ROC curve. (**d**–**f**), same as described in (**a**–**c**) for another example neuron.

**Figure 7:**
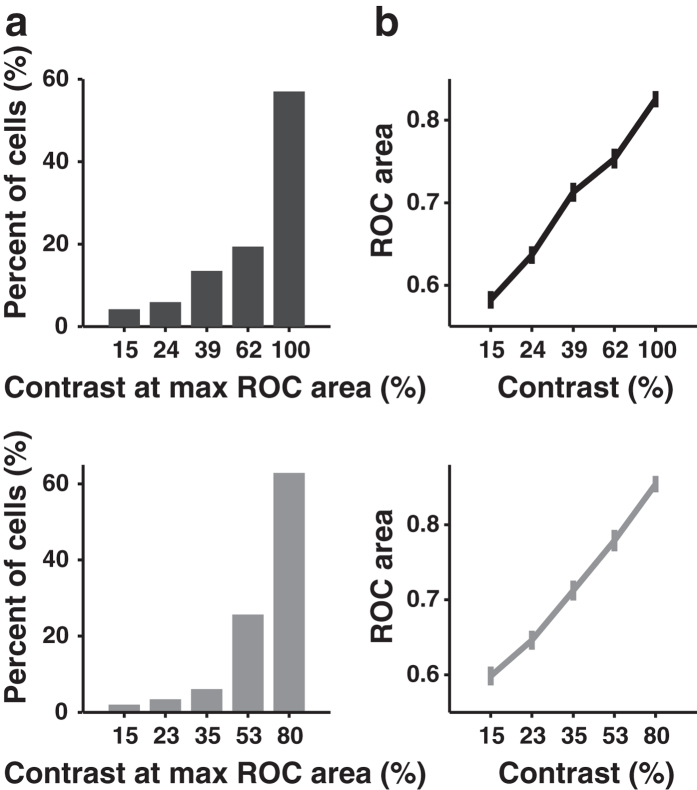
Analysis of neurometric functions of V1 neurons. (**a**) Distributions of the contrast at which the ROC area was maximum. Upper: the responses were measured using contrasts ranging from 15% to 100%, *n* = 237. Lower: the responses were measured with contrasts ranging from 15% to 80%, *n* = 148. (**b**) ROC area versus contrast, averaged over all neurons. Upper: the responses were measured using contrasts ranging from 15% to 100%, Spearman’s rank correlation coefficient *r* = 1, *P* = 0.008, *n* = 237. Lower: the responses were measured with contrasts ranging from 15% to 80%, Spearman’s rank correlation coefficient *r* = 1, *P* = 0.008, *n* = 148. Error bars, s.e.m.

Neuronal discriminability can be influenced by the variance as well as the mean of the responses^42^. Previous studies estimated the capacity of cortical neurons to discriminate visual stimuli by computing a neurometric function^42^,^43^,^44^ based on signal detection theory^39^. By a similar approach, we used the response distributions for preferred and orthogonal orientations at each contrast to construct a receiver operating characteristic (ROC) curve, in which the probability of the response to the preferred orientation exceeded a given criterion was plotted against that to the orthogonal orientation (Fig. 6). The area under the ROC curve (ROC area) is a nonparamatric measure of the probability that an ideal observer can correctly perform stimulus discrimination^39^,^44^. For the example cell shown in Fig. 6a, the distributions of responses to preferred and orthogonal orientations were more separated at higher contrast, leading to contrast-dependent increase in the ROC area (Fig. 6b,c). In Fig. 6d, the response distributions of the neuron were most separated at the second highest contrast so that the neurometric performance exhibited a non-monotonic dependence on contrast (Fig. 6e,f). We found that V1 neurons exhibited a variety shape of neurometric functions (Supplementary Fig. S3). For each neurometric function, we computed the contrast at which the ROC area reached the peak. Figure 7a shows the distribution of this peak contrast, the majority of which was at the highest contrast in the stimuli. The mean values of the maximum ROC area for those cells whose peak contrasts were at the highest contrast and those cells whose peak contrasts were at lower contrasts were both larger than 0.85 (Supplementary Fig. S4). Over the population, the ROC area increased monotonically with stimulus contrast (*n* = 237 and 148 for the two types of multiple contrast conditions, Fig. 7b). Taken together, the results indicated that the capacity of V1 neurons to discriminate two orthogonal orientations increased with stimulus contrast.

**Figure 8:**
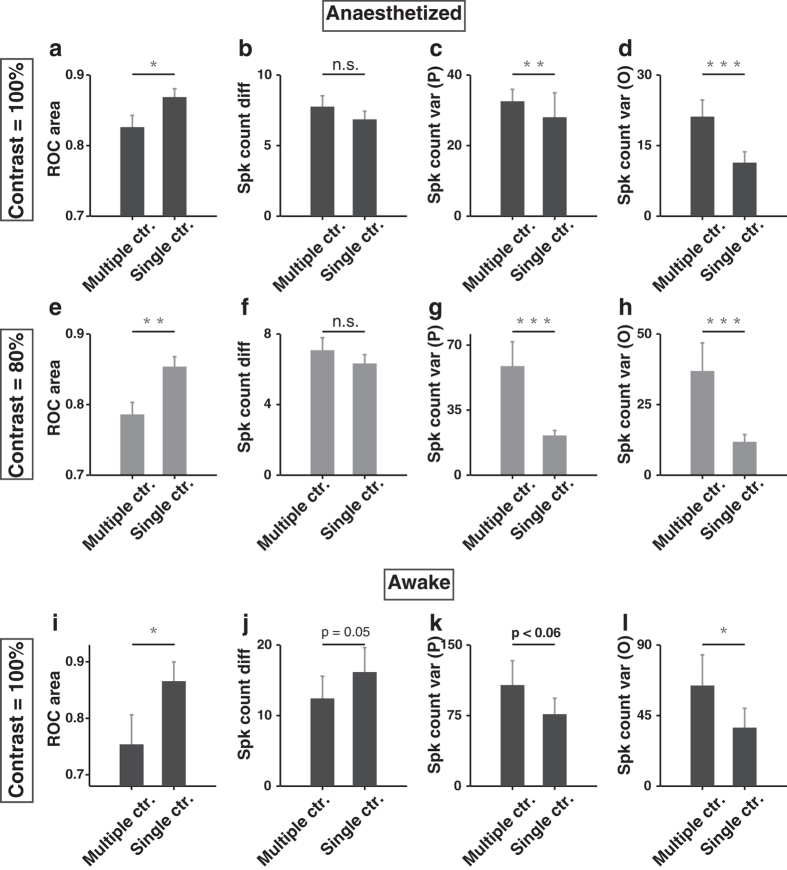
Comparison of ROC area and neural responses to high contrast stimuli between the single and the multiple contrast conditions. (**a**) ROC area at 100% contrast was significantly higher in the single than in the multiple contrast condition in anaesthetized mice. *P* = 0.01, *n* = 87, Wilcoxon signed rank test. (**b**) Response difference between preferred and orthogonal orientations at 100% contrast for the single and the multiple contrast conditions in anaesthetized mice. Spike count in each trial was computed as the number of spikes during the 2 s of the drifting grating. *P* = 0.15, *n* = 87, Wilcoxon signed rank test. (**c**,**d**) Response variance to preferred (P) or orthogonal (O) orientation at 100% contrast was significantly smaller in the single than in the multiple contrast condition in anaesthetized mice. *P* = 0.002 and 4.4 × 10^−7^, respectively, *n* = 87, Wilcoxon signed rank test. (**e**–**h**) Results for responses measured at 80% contrast in anaesthetized mice, similar as those described in (**a**–**d**). *n* = 92. (**i**–**l**) Results for responses measured at 100% contrast in awake mice, similar as those described in (**a**–**d**). *n* = 17. Error bars, s.e.m., *P < 0.05, **P < 0.01, ***P < 0.001, Wilcoxon signed rank test.

The behavioural experiments showed that the performance at high contrast could be adapted to the range of contrast in the stimuli (Fig. 1d). Is the capacity of V1 neurons to discriminate two high-contrast orientations also adapted to the contrast range in the stimuli? To address this issue, we compared the ROC area at high contrast (100% or 80%) between the single and the multiple contrast conditions (Methods). We found that the ROC area at 100% or 80% contrast was significantly larger in the single than in the multiple contrast condition (anaesthetized mice: *P* = 0.01 and *n* = 87 for 100% contrast in Fig. 8a, *P* = 0.005 and *n* = 92 for 80% contrast in Fig. 8e; awake mice: *P* = 0.02 and *n* = 17 for 100% contrast in Fig. 8i, Wilcoxon signed rank test). According to the signal detection theory, neural discriminability of two stimuli can be quantified by the difference of mean responses to the two stimuli divided by the square root of the mean response variance^39^,^45^. Larger difference between the means or smaller response variance could lead to smaller overlap between the distributions of responses to the two stimuli, resulting in higher discriminability. For anaesthetized mice, the response difference between preferred and orthogonal orientations at high contrast was not significantly different between the single and the multiple contrast conditions (*P* = 0.15 and *n* = 87 for 100% contrast, *P* = 0.65 and *n* = 92 for 80% contrast, Fig. 8b,f, Wilcoxon signed rank test); for awake mice, the response difference was larger in the single contrast condition (*P* = 0.05 and *n* = 17 for 100% contrast, Fig. 8j, Wilcoxon signed rank test). For both anaesthetized and awake mice, however, the response variance was significantly smaller in the single than in the multiple contrast condition (anaesthetized mice: *P* < 0.01, *n* = 87 and 92 for 100% and 80% contrast; awake mice: *P* < 0.06, *n* = 17 for 100% contrast, Fig. 8c,d,g,h,k,l, Wilcoxon signed rank test). The Fano factor, defined as the ratio of spike count variance to mean spike count, was not significantly different between the single and the multiple contrast conditions (Supplementary Fig. S5). Further analysis showed that, in anaesthetized mice, the mean firing rate to high contrast stimuli (100% or 80%) was lower in the single than in the multiple contrast condition (*P* = 8.4 × 10^−10^ and *n* = 87 for 100% contrast, *P* = 1.2 × 10^−5^ and *n* = 92 for 80% contrast, Wilcoxon signed rank test, Supplementary Fig. S6), suggesting that the adaptation effect is stronger in the single contrast condition. Thus, better neural discrimination of high-contrast oriented stimuli in the single contrast condition could be attributed to smaller response variance caused by stronger adaptation effect.

## Discussion

We examined the influence of contrast on the behavioural performance of mice to discriminate two orthogonal orientations using two task designs. In the 2AFC task, the performance increased with stimulus contrast. For both task designs, the performance at high contrast was better when the oriented stimuli were at a single contrast than at multiple contrasts. Physiological experiments showed that the ability of V1 neurons to discriminate two high-contrast orthogonal orientations was also higher in the single than in the multiple contrast condition, largely due to smaller response variance in the single contrast condition.

Previous psychophysical studies on human subjects showed that the performance of direction discrimination was dependent on stimulus contrast^14^,^15^. For orientation discrimination, although it has been shown that the discrimination threshold (i.e., the smallest orientation difference that can be discriminated) decreased with contrast^20^,^21^, there are few reports on how stimulus contrast affects the performance in discriminating two stimuli with a fixed orientation difference^32^ and whether the performance can be adapted to the range of contrast in the stimuli. Using a 2AFC task, we showed that the performance of mice to discriminate two orthogonal orientations increased with contrast. During a go/no-go task, when multiple contrasts were interleaved in the stimuli, the performance at the highest contrast tended to decrease, which was associated with an increase in the response bias. This result is likely due to the possibility that the low contrast stimuli in the multiple contrast condition may cause the animals to lower their threshold to respond^37^. Since the learning of go/no-go task was mainly due to decrease in FA rate (Supplementary Fig. S1), worse performance at high contrast in the go/no-go task may be explained not only by an increase in the response bias, but also by a general inability of the mice to withhold their responses. For the go/no-go task, the performance at the highest contrast in the multiple contrast condition could rapidly improve when the stimuli in previous trials were at the same high contrast (Supplementary Fig. S7), suggesting that the animal’s internal threshold to respond can rapidly adapt to the temporal context of stimulus contrast. Our results are also consistent with the notion that go/no-go tasks are vulnerable to changes in the animal’s response criterion^37^. In the 2AFC task design, both the target and the non-target stimuli were presented at the same time, and the animal was given two choices of responses at two locations. Since the animal must respond on every trial and was forced to indicate the presence of target in one location and non-target in another location, the 2AFC task design was not affected by the animal’s willingness to respond or response criterion^37^. Thus, behavioural results of the 2AFC task truly reflect the influence of stimulus contrast on orientation discrimination. Our results also point to the importance of task design for behavioural study of mouse vision.

We found that the response difference between preferred and orthogonal orientations in mouse V1 was also contrast dependent. The response difference averaged over all neurons increased with contrast, consistent with recent reports in ferret V1 and in layer 4 of mouse V1^11^,^12^. The ROC analysis also showed that the majority of V1 neurons exhibited monotonic neurometric functions, whose maximum ROC area was at the highest contrast. Thus, the contrast-dependent orientation discriminability of V1 neurons may contribute to the contrast-dependent behavioural performance observed in the 2AFC task.

When the stimulus set contained only a single high contrast, the behavioural performance of orientation discrimination was significantly better than that in the multiple contrast condition. Although the effect observed in the go/no-go task may be due to increase in the response bias in the multiple contrast condition (Supplementary Fig. S2), the effect observed in the 2AFC task can not be explained by changes in the animal’s internal response criterion. Reminiscent of the behavioural results (Fig. 1d), the orientation discriminability of V1 neurons at high contrast was significantly higher in the single than in the multiple contrast condition (Fig. 8a,e,i). Thus, the contrast-range dependent capacity of V1 neurons to discriminate high-contrast orientations may partly explain the behavioural result that performance at high contrast was better in the single than in the multiple contrast condition for the 2AFC task.

Previous studies have demonstrated that the contrast response functions of cortical neurons can be adjusted to match the prevailing mean contrast level in the environment^46^,^47^,^48^,^49^,^50^. At different levels of contrast adaptation, the contrast response curves of cortical neurons span the same response range but shift along the log-contrast axis. Such contrast gain control allows cortical neurons to use their limited response range to encode a large contrast range. Furthermore, adaptation to higher contrast causes larger decrease in the response^49^, and the reduced response is associated with reduced response variance^45^. In our study, we found that the variance of V1 responses to preferred or orthogonal orientation at high contrast (80% or 100%) was substantially smaller in the single than in the multiple contrast condition, whereas the response difference between preferred and orthogonal orientations was similar in the two conditions (Fig. 8). The smaller response variance in the single contrast condition could be due to stronger adaptation effect in the single than in the multiple contrast condition (Supplementary Fig. S6). Since neural discriminability can be quantified by the difference of mean responses to two stimuli divided by the square root of the mean response variance^39^,^45^, the decrease in response variance can presumably cause decrease in the overlap between the distributions of responses to the two orthogonal orientations, leading to a higher degree of orientation discriminability in the single contrast condition.

## Methods

All procedures complied with the guidelines of the Animal Advisory Committee at the Shanghai Institutes for Biological Sciences, and the protocol was approved by the Animal Care and Use Committee at the Institute of Neuroscience, Chinese Academy of Sciences.

### Animals and surgery

For headplate implant, mice (C57BL/6) were anaesthetized with an intraperitoneal injection of the mixture of ketamine (200 mg/kg) and xylazine (10 mg/kg). The animal was head-fixed in a stereotaxic apparatus. Body temperature was maintained at 37 °C with a heating blanket. A thin layer of tissue adhesive (Vetbond, 3 M) was applied to the skull and a stainless-steel headplate was cemented to the skull with dental acrylic. Antiphlogistic drug (ceftriaxone sodium, 2 mg/kg, intramuscular injection) was administered. Mice were allowed to recover with food and water *ad libitum* at least one week before behavioural training of the go/no-go task.

We recorded from the monocular zone of V1 in both anaesthetized and awake mice (C57BL/6). Details for surgery are described in the Supplementary Note.

### Behaviour

Mice were restricted from free access to water before the behaviour training. Eight and fourteen mice (C57BL/6, male) were used in the 2AFC and go/no-go task, respectively.

The 2AFC behavioural experiments were conducted in a custom-designed chamber (36 × 17 × 29 cm, L × W × H), with three ports positioned along the front wall facing an LCD monitor^34^. The chamber was separated into three connected areas by two dividers. Nose-poke into each port was detected by the interruption of an infrared beam. Mice were trained to perform the task using the following steps^51^. In step 1 (2–3 d), the center port was blocked, and mice were rewarded with water (10 μl) by poking their noses into the two side ports in an alternating manner. The delivery of water was controlled by a solenoid valve (SMC). In step 2 (6–8 d), with the right port blocked by a barrier, mice initiated the presentation of target stimulus (a vertically oriented static grating) on the left side of the monitor by a nose poke into the center port, and learned to receive water reward from the left port. After mice receiving ∼1.5 ml of water within one session, the barrier was changed to the left port. Poking into the center port triggered the presentation of target stimulus on the right side, and mice learned to receive water reward from the right port. In step 3 (∼three weeks), with all ports open, mice initiated a trial by poking the center port, and the target stimulus was presented on one side of the monitor (the non-target was not presented). Choosing the port at the side the target was presented was rewarded by water (4–5 μl), and choosing the incorrect port was followed by a timeout period (8 s). The stimulus disappeared after the mice made the choice. Mice advanced to the next step after reaching a correct rate of ∼80% for three consecutive sessions. In step 4 (2–3 d), the target and the non-target stimulus (a horizontally oriented static grating), both at 100% contrast, were presented on two sides of the monitor, and mice learned to choose the port at the side the target was presented. In step 5, we gradually changed the single contrast used in step 4 to multiple contrasts by adding one or two contrasts at a time until a total of five contrasts (ranging from 15% to100% in log step) were used for stimulus set in the same session.

For the go/no-go task, mice went through the following steps of training. In step 1 (a week), mice were handled daily for ∼5 min, after which ∼1 ml of water was given. In step 2 (2–4 d), the head-fixed mouse sat in a circular plastic tube and learned to lick from a lickspout close to the mouth. In step 3 (2–6 d), mice were trained to associate licking with the presentation of target stimulus (a horizontally oriented grating drifting upward for 3 s). The period 1 to 3 s after stimulus onset was the response window, during which licking resulted in an immediate water reward of 5–7 μl. In step 4, mice were trained to discriminate between the target and the non-target stimulus (vertically oriented grating drifting leftward). The duration of each stimulus was 3 s. A blank gray screen (30 cd/m^2^) was presented during the inter-stimulus interval (ISI) at a default value of 4 s. Licking during the response window of target presentation resulted in water reward. Licking during the response window of non-target presentation was punished by a brief and mild air puff to the cheek. During ISI, licking within the first 1 s was not punished, but licking afterwards triggered the air puff and led to the immediate start of a timeout period, which was another 4-s ISI. When there was no lick during the ISI or the accumulated ISI exceeded 20 s, ISI stopped and another trial of target or non-target stimulus was presented. Mice were first trained with stimuli at a single contrast of 100% until *d*′* *> 1 for at least three consecutive sessions. We next gradually changed the single contrast to multiple contrasts by adding one or two contrasts at a time until a total of five contrasts (ranging from 15% to 100%, or from 15% to 80% in log step) were used for stimulus set in the same session.

In the multiple contrast condition, each block included four trials of the same contrast (see Supplementary Note for details). The performance in the multiple contrast condition was measured for 3–4 sessions (2AFC task) or 5–12 sessions (go/no-go task). After measuring the mice’s performance with multiple contrasts, we re-measured their performance at a single contrast of 100% or 80% for 2–3 sessions (2AFC task) or 5–7 sessions (go/no-go task).

### Visual stimulation

For the 2AFC task, visual stimuli (static sinusoidal grating, 46° × 46°, 0.039 cycle/°, assuming that the mouse was at the port facing the stimulus) were presented on a 17′′ LCD monitor (Dell E1713S, mean luminance 30 cd/m^2^, refresh rate 60 Hz). For the go/no-go task, visual stimuli (drifting sinusoidal grating, 74° × 74°, 0.025 cycle/°, 1.5 Hz) were presented on a 19′′ CRT monitor (ViewSonic P225f, minimum, mean, and maximum luminance were 0.09, 30, and 58.9 cd/m^2^, respectively, refresh rate 60 Hz) placed 25 cm away from the animal’s left eye. Gamma correction was used to calibrate the monitors.

For electrophysiological recordings, visual stimuli (53° × 53° for anaesthetized mice and 66° × 66° for awake mice, sinusoidal grating, 0.025 cycle/°, 1.5 Hz) covering the receptive fields of all recorded units were presented to the contralateral eye on the same CRF monitor described above. For the multiple contrast condition, drifting gratings at 12 orientations (spaced at 30°) and 5 contrast levels were presented in a random sequence. The 5 contrast levels were [15%, 23%, 35%, 53%, 80%] or [15%, 24% 39%, 62%, 100%], similar to those in the behavioural experiments. For the single contrast condition, drifting gratings (contrast at 100% or 80%) at 12 orientations (spaced at 30°) were presented in a random sequence. Each orientation and each contrast was presented for 10 trials. Each trial started with 0.5 s of gray screen (30 cd/m^2^), followed by 0.3 s of the first frame of grating and 2 s of the drifting grating.

### Electrophysiology

Recordings were made with multi-site silicon probes (A1 × 16–3 mm-50-177 or A2 × 2-tet-3 mm-150-150-121, NeuroNexus Technologies) (see Supplementary Note for details).

### Analysis of behavior

To quantify the correct rate for each contrast in the 2AFC task, we calculated the percentage of rightward choices for those trials in which the target was presented on the right side and the percentage of leftward choices for those trials in which the target was presented on the left side, and averaged the two percentages. For the single contrast condition or the multiple contrast condition, we averaged the correct rates of all sessions for each mouse.

For the go/no-go task, hit rate was defined as the ratio between the number of hits and the total number of targets. False alarm (FA) rate was defined as the ratio between the number of FAs and the total number of non-targets. Miss rate was defined as the ratio between the number of misses and the total number of targets. Response bias was quantified as: (number of hits + number of FAs)/(total number of stimuli). Discriminability (*d*′) was given by *d*′ = norminv(hit rate)—norminv(false alarm rate), where norminv() is the inverse function of the cumulative Gaussian distribution^39^. For the initial training with a single contrast of 100%, discriminability before training was computed as *d*′ averaged over the first 3 sessions, and discriminability after training was computed as *d*′ averaged over the last 3 sessions. For the multiple contrast condition or the single contrast condition measured after the multiple contrast condition, discriminability was computed as *d*′ averaged over the last 3 sessions. Lick efficiency was defined as the ratio between the number of licks within the response window and the total number of licks in a target trial^40^.

### Analysis of neuronal responses

Responses during the 2 s presentation of grating were averaged for each orientation and contrast. The spontaneous response was computed as the mean firing rate during the last 0.3 s of the gray screen presented before all trials of grating stimuli. For the multiple contrast condition, we first identified the stimulus at which the response was maximum, then extracted the responses to different orientations at the contrast of this stimulus and fitted the orientation tuning with the sum of two Gaussian functions:


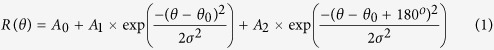


where *R*(*θ*) is the response at orientation *θ*, and *A*_0_, *A*_1_, *A*_2_, *θ*_0_, and 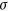 are free parameters. Orientation tuning measured in the single contrast condition was also fitted with the same function. The fitting error was computed as^31^:


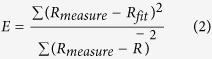


where 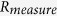 and 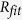 are the measured and fitted responses, respectively, and 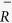 is the measured response averaged over all orientations. We also quantified the degree of orientation selectivity with a global measure of orientation selectivity index (OSI)^52^,^53^:


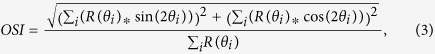


where 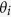 is the angle of the direction of the drifting grating and 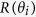 is the response at angle 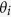. Only cells whose evoked responses were significantly larger than the spontaneous responses (determined by a Kruskal-Wallis test, *P* < 0.01), OSI > 0.08^54^, and fitting error < 0.5^31^ were included in the analysis. The cells included in the analysis were from 30 anaesthetized and 38 awake mice (see Supplementary Note for details).

The preferred orientation was defined as the orientation at which the response was maximum. We computed the response difference between preferred and orthogonal orientations (preferred orientation + 90°) for each contrast level. To quantify the degree of monotonic dependence of response difference on contrast, we computed a dimensionless monotonicity index (MI)^5^:


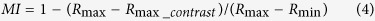


where *R*_max_contrast_ is the response difference at the maximal contrast tested, 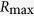 and 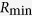 are the maximal and minimal response difference.

Neurometric functions are computed using the distributions of single-trial responses to preferred and orthogonal orientations^44^ (see Supplementary Note for details).

## Additional Information

**How to cite this article**: Long, M. *et al.* Contrast-dependent orientation discrimination in the mouse. *Sci. Rep.* **5**, 15830; doi: 10.1038/srep15830 (2015).

## Supplementary Material

Supplementary Information

## References

[b1] Movshon J. A. & Tolhurst D. J. Proceedings: On the response linearity of neurones in cat visual cortex. J. Physiol. 249, 56P–57P (1975). 1151880

[b2] Albrecht D. G. & Hamilton D. B. Striate cortex of monkey and cat: contrast response function. J. Neurophysiol. 48, 217–237 (1982). 7119846 10.1152/jn.1982.48.1.217

[b3] Gao E., DeAngelis G. C. & Burkhalter A. Parallel input channels to mouse primary visual cortex. J. Neurosci. 30, 5912–5926 (2010). 20427651 10.1523/JNEUROSCI.6456-09.2010 PMC3129003

[b4] Li C. Y. & Creutzfeldt O. The representation of contrast and other stimulus parameters by single neurons in area 17 of the cat. Pflugers Arch. 401, 304–314 (1984). 6473083 10.1007/BF00582601

[b5] Ledgeway T., Zhan C., Johnson A. P., Song Y. & Baker C. L. Jr. The direction-selective contrast response of area 18 neurons is different for first- and second-order motion. Visual Neurosci. 22, 87–99 (2005). 10.1017/S0952523805221120 15842744

[b6] Peterson M. R., Li B. & Freeman R. D. Direction selectivity of neurons in the striate cortex increases as stimulus contrast is decreased. J. Neurophysiol. 95, 2705–2712 (2006). 16306177 10.1152/jn.00885.2005

[b7] Sceniak M. P., Ringach D. L., Hawken M. J. & Shapley R. Contrast’s effect on spatial summation by macaque V1 neurons. Nature Neurosci. 2, 733–739 (1999). 10412063 10.1038/11197

[b8] Sceniak M. P., Hawken M. J. & Shapley R. Contrast-dependent changes in spatial frequency tuning of macaque V1 neurons: effects of a changing receptive field size. J. Neurophysiol. 88, 1363–1373 (2002). 12205157 10.1152/jn.2002.88.3.1363

[b9] Chen K., Song X. M. & Li C. Y. Contrast-dependent variations in the excitatory classical receptive field and suppressive nonclassical receptive field of cat primary visual cortex. Cereb. Cortex 23, 283–292 (2013). 22302117 10.1093/cercor/bhs012

[b10] Nienborg H. *et al.* Contrast dependence and differential contributions from somatostatin- and parvalbumin-expressing neurons to spatial integration in mouse V1. J. Neurosci. 33, 11145–11154 (2013). 23825418 10.1523/JNEUROSCI.5320-12.2013 PMC3718383

[b11] Alitto H. J. & Usrey W. M. Influence of contrast on orientation and temporal frequency tuning in ferret primary visual cortex. J. Neurophysiol. 91, 2797–2808 (2004). 14762157 10.1152/jn.00943.2003

[b12] Li Y. T. *et al.* Broadening of inhibitory tuning underlies contrast-dependent sharpening of orientation selectivity in mouse visual cortex. J. Neurosci. 32, 16466–16477 (2012). 23152629 10.1523/JNEUROSCI.3221-12.2012 PMC3548445

[b13] Johnson E. N., Hawken M. J. & Shapley R. The orientation selectivity of color-responsive neurons in macaque V1. J. Neurosci. 28, 8096–8106 (2008). 18685034 10.1523/JNEUROSCI.1404-08.2008 PMC2896204

[b14] Derrington A. M. & Goddard P. A. Failure of motion discrimination at high contrasts: evidence for saturation. Vision Res. 29, 1767–1776 (1989). 2631398 10.1016/0042-6989(89)90159-4

[b15] Cleary R. Contrast dependence of short-range apparent motion. Vision Res. 30, 463–478 (1990). 2336804 10.1016/0042-6989(90)90087-2

[b16] Hawken M. J., Gegenfurtner K. R. & Tang C. Contrast dependence of colour and luminance motion mechanisms in human vision. Nature 367, 268–270 (1994). 8121491 10.1038/367268a0

[b17] Stone L. S. & Thompson P. Human speed perception is contrast dependent. Vision Res. 32, 1535–1549 (1992). 1455726 10.1016/0042-6989(92)90209-2

[b18] Smith B. G. & Thomas J. P. Why are some spatial discriminations independent of contrast? J. Opt. Soc. Am. A 6, 713–724 (1989). 2723847 10.1364/josaa.6.000713

[b19] Thomas J. P. & Olzak L. A. Contrast gain control and fine spatial discriminations. J. Opt. Soc. Am. A 14, 2392–2405 (1997). 10.1364/josaa.14.002392 9291609

[b20] Mareschal I. & Shapley R. M. Effects of contrast and size on orientation discrimination. Vision Res. 44, 57–67 (2004). 14599571 10.1016/j.visres.2003.07.009

[b21] Skottun B. C., Bradley A., Sclar G., Ohzawa I. & Freeman R. D. The effects of contrast on visual orientation and spatial frequency discrimination: a comparison of single cells and behavior. J. Neurophysiol. 57, 773–786 (1987). 3559701 10.1152/jn.1987.57.3.773

[b22] Mareschal I., Andrew Henrie J. & Shapley R. M. A psychophysical correlate of contrast dependent changes in receptive field properties. Vision Res. 42, 1879–1887 (2002). 12128018 10.1016/s0042-6989(02)00099-8

[b23] Huberman A. D. & Niell C. M. What can mice tell us about how vision works? Trends Neurosci. 34, 464–473 (2011). 21840069 10.1016/j.tins.2011.07.002 PMC3371366

[b24] Niell C. M. & Stryker M. P. Highly selective receptive fields in mouse visual cortex. J. Neurosci. 28, 7520–7536 (2008). 18650330 10.1523/JNEUROSCI.0623-08.2008 PMC3040721

[b25] Liu B. H. *et al.* Visual receptive field structure of cortical inhibitory neurons revealed by two-photon imaging guided recording. J. Neurosci. 29, 10520–10532 (2009). 19710305 10.1523/JNEUROSCI.1915-09.2009 PMC2779138

[b26] Stroud A. C., Ledue E. E. & Crowder N. A. Orientation specificity of contrast adaptation in mouse primary visual cortex. J. Neurophysiol. 108, 1381–1391 (2012). 22696541 10.1152/jn.01148.2011

[b27] LeDue E. E., King J. L., Stover K. R. & Crowder N. A. Spatiotemporal specificity of contrast adaptation in mouse primary visual cortex. Front. Neural Circuits 7, 154 (2013). 24106461 10.3389/fncir.2013.00154 PMC3789212

[b28] Douglas R. M., Neve A., Quittenbaum J. P., Alam N. M. & Prusky G. T. Perception of visual motion coherence by rats and mice. Vision Res. 46, 2842–2847 (2006). 16647739 10.1016/j.visres.2006.02.025

[b29] Reuter J. H. Tilt discrimination in the mouse. Behav. Brain Res. 24, 81–84 (1987). 3580117 10.1016/0166-4328(87)90038-6

[b30] Andermann M. L., Kerlin A. M. & Reid R. C. Chronic cellular imaging of mouse visual cortex during operant behavior and passive viewing. Front. Cell. Neurosci. 4, 3 (2010). 20407583 10.3389/fncel.2010.00003 PMC2854571

[b31] Lee S. H. *et al.* Activation of specific interneurons improves V1 feature selectivity and visual perception. Nature 488, 379–383 (2012). 22878719 10.1038/nature11312 PMC3422431

[b32] Pinto L. *et al.* Fast modulation of visual perception by basal forebrain cholinergic neurons. Nature Neurosci. 16, 1857–1863 (2013). 24162654 10.1038/nn.3552 PMC4201942

[b33] Glickfeld L. L., Histed M. H. & Maunsell J. H. Mouse primary visual cortex is used to detect both orientation and contrast changes. J. Neurosci. 33, 19416–19422 (2013). 24336708 10.1523/JNEUROSCI.3560-13.2013 PMC3858618

[b34] Busse L. *et al.* The detection of visual contrast in the behaving mouse. J. Neurosci. 31, 11351–11361 (2011). 21813694 10.1523/JNEUROSCI.6689-10.2011 PMC6623377

[b35] Histed M. H., Carvalho L. A. & Maunsell J. H. Psychophysical measurement of contrast sensitivity in the behaving mouse. J. Neurophysiol. 107, 758–765 (2012). 22049334 10.1152/jn.00609.2011 PMC3289478

[b36] Bennett C., Arroyo S. & Hestrin S. Subthreshold mechanisms underlying state-dependent modulation of visual responses. Neuron 80, 350–357 (2013). 24139040 10.1016/j.neuron.2013.08.007 PMC3806653

[b37] Carandini M. & Churchland A. K. Probing perceptual decisions in rodents. Nature Neurosci. 16, 824–831 (2013). 23799475 10.1038/nn.3410 PMC4105200

[b38] O’Connor D. H. *et al.* Neural coding during active somatosensation revealed using illusory touch. Nature Neurosci. 16, 958–965 (2013). 23727820 10.1038/nn.3419 PMC3695000

[b39] Green D. M. & Swets J. A. Signal detection theory and psychophysics (Wiley, New York, 1966).

[b40] Komiyama T. *et al.* Learning-related fine-scale specificity imaged in motor cortex circuits of behaving mice. Nature 464, 1182–1186 (2010). 20376005 10.1038/nature08897

[b41] Sani I., Santandrea E., Golzar A., Morrone M. C. & Chelazzi L. Selective tuning for contrast in macaque area V4. J. Neurosci. 33, 18583–18596 (2013). 24259580 10.1523/JNEUROSCI.3465-13.2013 PMC6618802

[b42] Tolhurst D. J., Movshon J. A. & Dean A. F. The statistical reliability of signals in single neurons in cat and monkey visual cortex. Vision Res. 23, 775–785 (1983). 6623937 10.1016/0042-6989(83)90200-6

[b43] Britten K. H., Shadlen M. N., Newsome W. T. & Movshon J. A. The analysis of visual motion: a comparison of neuronal and psychophysical performance. J. Neurosci. 12, 4745–4765 (1992). 1464765 10.1523/JNEUROSCI.12-12-04745.1992 PMC6575768

[b44] Parker A. J. & Newsome W. T. Sense and the single neuron: probing the physiology of perception. Annu. Rev. Neurosci. 21, 227–277 (1998). 9530497 10.1146/annurev.neuro.21.1.227

[b45] Müller J. R., Metha A. B., Krauskopf J. & Lennie P. Rapid adaptation in visual cortex to the structure of images. Science 285, 1405–1408 (1999). 10464100 10.1126/science.285.5432.1405

[b46] Ohzawa I., Sclar G. & Freeman R. D. Contrast gain control in the cat visual cortex. Nature 298, 266–268 (1982). 7088176 10.1038/298266a0

[b47] Ohzawa I., Sclar G. & Freeman R. D. Contrast gain control in the cat’s visual system. J. Neurophysiol. 54, 651–667 (1985). 4045542 10.1152/jn.1985.54.3.651

[b48] Sclar G., Lennie P. & DePriest D. D. Contrast adaptation in striate cortex of macaque. Vision Res. 29, 747–755 (1989). 2623819 10.1016/0042-6989(89)90087-4

[b49] Albrecht D. G., Farrar S. B. & Hamilton D. B. Spatial contrast adaptation characteristics of neurones recorded in the cat’s visual cortex. J. Physiol. 347, 713–739 (1984). 6707974 10.1113/jphysiol.1984.sp015092 PMC1199473

[b50] Hu M. & Wang Y. Rapid dynamics of contrast responses in the cat primary visual cortex. PLoS One 6, e25410 (2011). 21998655 10.1371/journal.pone.0025410 PMC3187764

[b51] Stubblefield E. A., Costabile J. D. & Felsen G. Optogenetic investigation of the role of the superior colliculus in orienting movements. Behav. Brain Res. 255, 55–63 (2013). 23643689 10.1016/j.bbr.2013.04.040 PMC3796036

[b52] Dragoi V., Sharma J. & Sur M. Adaptation-induced plasticity of orientation tuning in adult visual cortex. Neuron 28, 287–298 (2000). 11087001 10.1016/s0896-6273(00)00103-3

[b53] Ringach D. L., Shapley R. M. & Hawken M. J. Orientation selectivity in macaque V1: diversity and laminar dependence. J. Neurosci. 22, 5639–5651 (2002). 12097515 10.1523/JNEUROSCI.22-13-05639.2002 PMC6758222

[b54] Xu X., Ichida J., Shostak Y., Bonds A. B. & Casagrande V. A. Are primate lateral geniculate nucleus (LGN) cells really sensitive to orientation or direction? Visual Neurosci. 19, 97–108 (2002). 10.1017/s0952523802191097 12180863

