# Supplementary material for: Contrast-dependent orientation discrimination in the mouse
Source: Sci Rep. 2015 Oct 29;5:15830. doi: 10.1038/srep15830 (PMC4625186; doi:10.1038/srep15830)
Supplement: Supplementary Information [file srep15830-s1.pdf]

Supplementary Information

Contrast-dependent orientation discrimination in the mouse

Minghai Long[1,2], Weiqian Jiang[1,2], Dechen Liu[1,2] & Haishan Yao[1,*]

[1]Institute of Neuroscience and State Key Laboratory of Neuroscience, Shanghai Institutes for Biological Sciences, Chinese Academy of Sciences, Shanghai 200031, China

[2]University of Chinese Academy of Sciences, Shanghai 200031, China

This Supplementary Information contains:

Supplementary Note

Supplementary Figures S1-S7

Supplementary Note

1. Surgery

For the anaesthetized experiments, the mice were sedated with an intraperioneal injection of chlorprothixene (4 mg/kg) and anaesthetized with urethane (1.2 g/kg). Dexamethasone (2 mg/kg) and atropine (0.3 mg/kg) were injected subcutaneously to reduce edema and secretions. We monitored the toe-pinch reflex of the animal and supplemented additional urethane (0.2 - 0.3 g/kg) as needed. For the awake experiments, the headplate of the mouse was fixed to a holder attached to the stereotaxic apparatus and the mouse's body was restricted in a circular plastic tube. Following anaesthesia with isoflurane (~ 1%), a craniotomy (~ 1 mm diameter) was made above V1, and the exposed cortex was protected with a silicone elastomer (Kwik-Cast, World Precision Instruments). The mouse was let to recover from the anaesthesia for at least 3 hours, after which the animal was head-fixed to a holder via the headplate. The dura was removed before the recordings. The recordings lasted 3 - 5 hours in anaesthetized mice and 2 - 3 hours in awake mice. After the recordings, the mice were euthanized by an overdose of pentobarbital (0.5 g/kg) followed by cervical dislocation.

2. Behaviour

In the multiple contrast condition, each block included four trials of the same contrast. For the 2AFC task, each block included two trials in which the target was on the left side and two trials in which the target was on the right side, and the sequence of the four trials within each block as well as the contrasts in different blocks were randomized. For the go/no-go task, the four trials in each block included two targets and two non-targets of the same contrast, and the contrasts in different blocks as well as the sequence of stimuli within each block were randomized.

For the multiple contrast condition in the 2AFC task, the contrasts ranged from 15% to 100%. For the

go/no-go task, the multiple contrasts ranged from 15% to 100% or from 15% to 80%, and all animals were trained on both versions of the multiple contrast condition.

In each behavioural session, each mouse performed 390 - 560 trials (average = 439 ± 35, mean ± s.d.) for the 2AFC task and 230 - 590 trials (average = 365 ± 48, mean ± s.d.) for the go/no-go task.

3. Electrophysiology

The neural signals were amplified at 30 kHz and filtered with a Cerebus 64-channel system (Blackrock microsystem). We band-pass filtered the signals at 0.25 to 7.5 kHz and set a threshold at 4 s.d. of the background noise to detect spike waveforms. Spikes were sorted offline with the Offline Sorter (Plexon Inc.) using cluster analysis of principal component amplitudes. Spike clusters were considered to be single units if their interspike interval was > 1 ms and $P < 0.05$ for multivariate ANOVA tests on clusters. To determine whether a single neuron was recorded by more than one site in the electrode, we computed correlation coefficients (binned at 1 ms) between all pair-wise combinations of units in the same recording. Those pairs with a correlation coefficient > 0.1 were considered to contain duplicate units, and the unit with the lower firing rate in the pair was discarded[1].

4. Analysis of neuronal responses

In **Fig. 5** and **Fig. 7**, 188 neurons from anaesthetized mice and 49 neurons from awake mice were included for the responses measured with contrasts ranging from 15% to 100%, and 148 neurons from anaesthetized mice were included for the responses measured with contrasts ranging from 15% to 80%. In **Fig. 8**, 87 and 92 neurons from anesthetized mice were included for the responses at 100% and 80% contrast, respectively, and 17 neurons from awake mice were included for the responses at 100% contrast.

For each contrast, we compiled a receiver operating characteristic (ROC) curve for the pair of response distributions using a range of 30 criterion values spanning from the minimal to the maximal

response[2]. The probability of correct discrimination was calculated as the area under the ROC curve (ROC area). After performing this analysis for all contrast levels, we generated a neurometric function for each neuron. When we compared the ROC area at high contrast (100% or 80%) between the single and the multiple contrast conditions, we only used those cells in which both conditions were tested in the same cell.

References

1 Goard, M. & Dan, Y. Basal forebrain activation enhances cortical coding of natural scenes. *Nature Neurosci.* **12**, 1444-1449 (2009).

2 Britten, K. H., Shadlen, M. N., Newsome, W. T. & Movshon, J. A. The analysis of visual motion: a comparison of neuronal and psychophysical performance. *J. Neurosci.* **12**, 4745-4765 (1992).

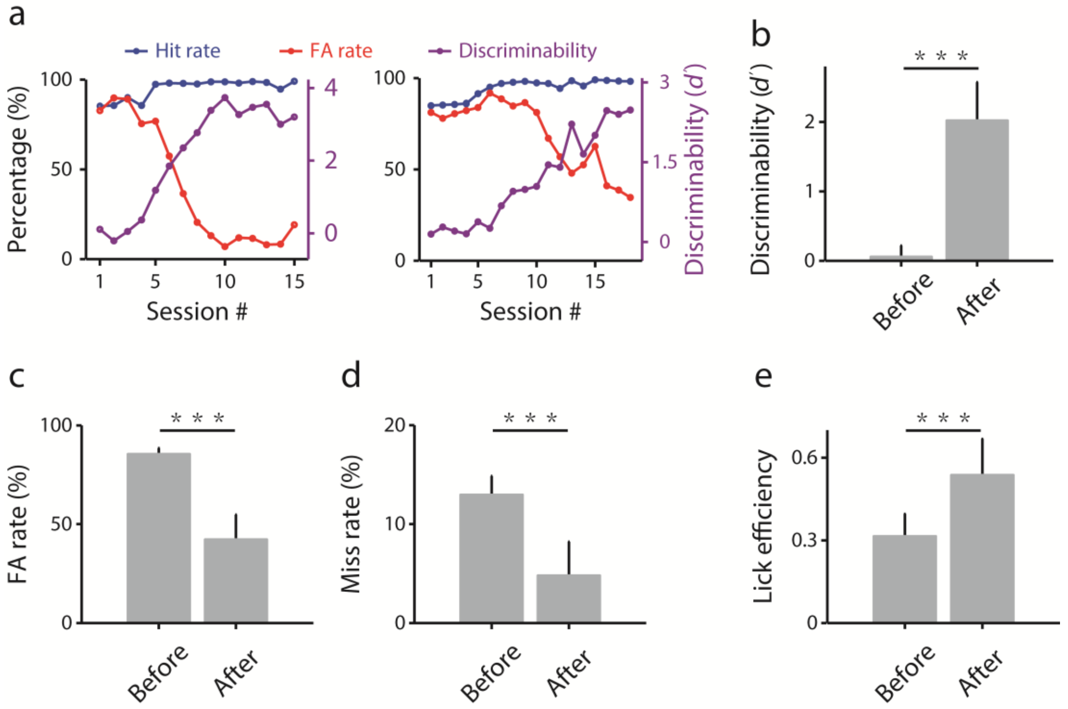

Figure S1. Mice learned to discriminate orientations in a go/no-go task after training. (a) Hit rate, FA rate, and discriminability over sessions for two example mice. (b) Comparison of discriminability before and after training. (c) Comparison of FA rate before and after training. (d) Comparison of miss rate before and after training. (e) Comparison of lick efficiency before and after training. The stimuli were at a single contrast of 100%. *n* = 14 mice. Error bars, s.d., ***, $P < 0.001$, Wilcoxon signed rank test.

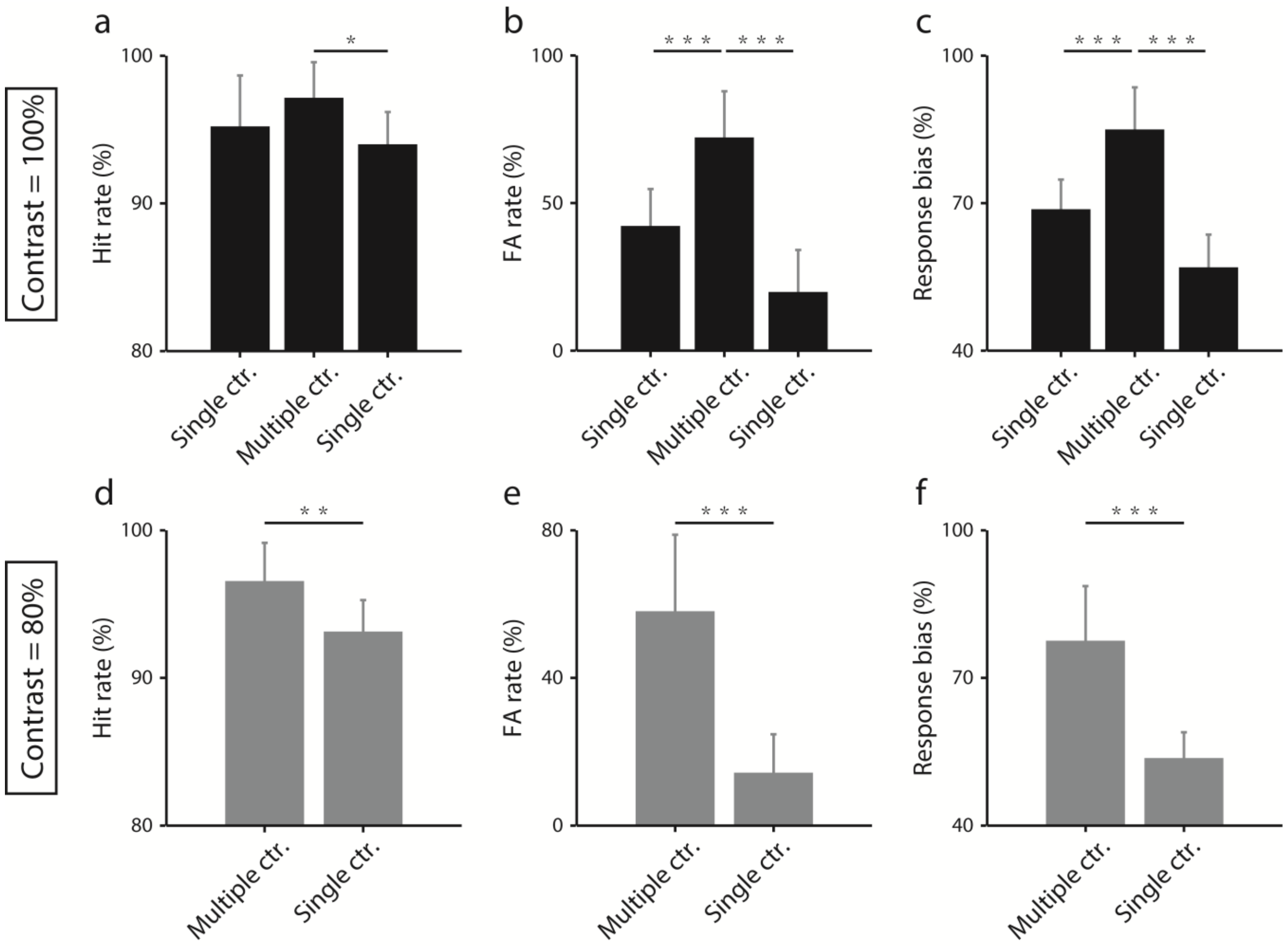

Figure S2. Comparison of behavioural performance at high contrast between the single and the multiple contrast conditions for the go/no-go task. (a) Hit rate at 100% contrast was above 90% for both the single and the multiple contrast conditions. The single contrast condition was tested both before (left bar) and after (right bar) the multiple contrast condition. (b) FA rate at 100% contrast was significantly lower in the single than in the multiple contrast condition. (c) Response bias at 100% contrast was significantly lower in the single than in the multiple contrast condition. (d-f), Hit rate, FA rate, and response bias for 80% contrast in the single and the multiple contrast conditions, similar as those described in (a-c). The single contrast condition was tested after the multiple contrast condition. Error bars, s.d., n = 14 mice. *, $P < 0.05$, **, $P < 0.01$, ***, $P < 0.001$, Wilcoxon signed rank test.

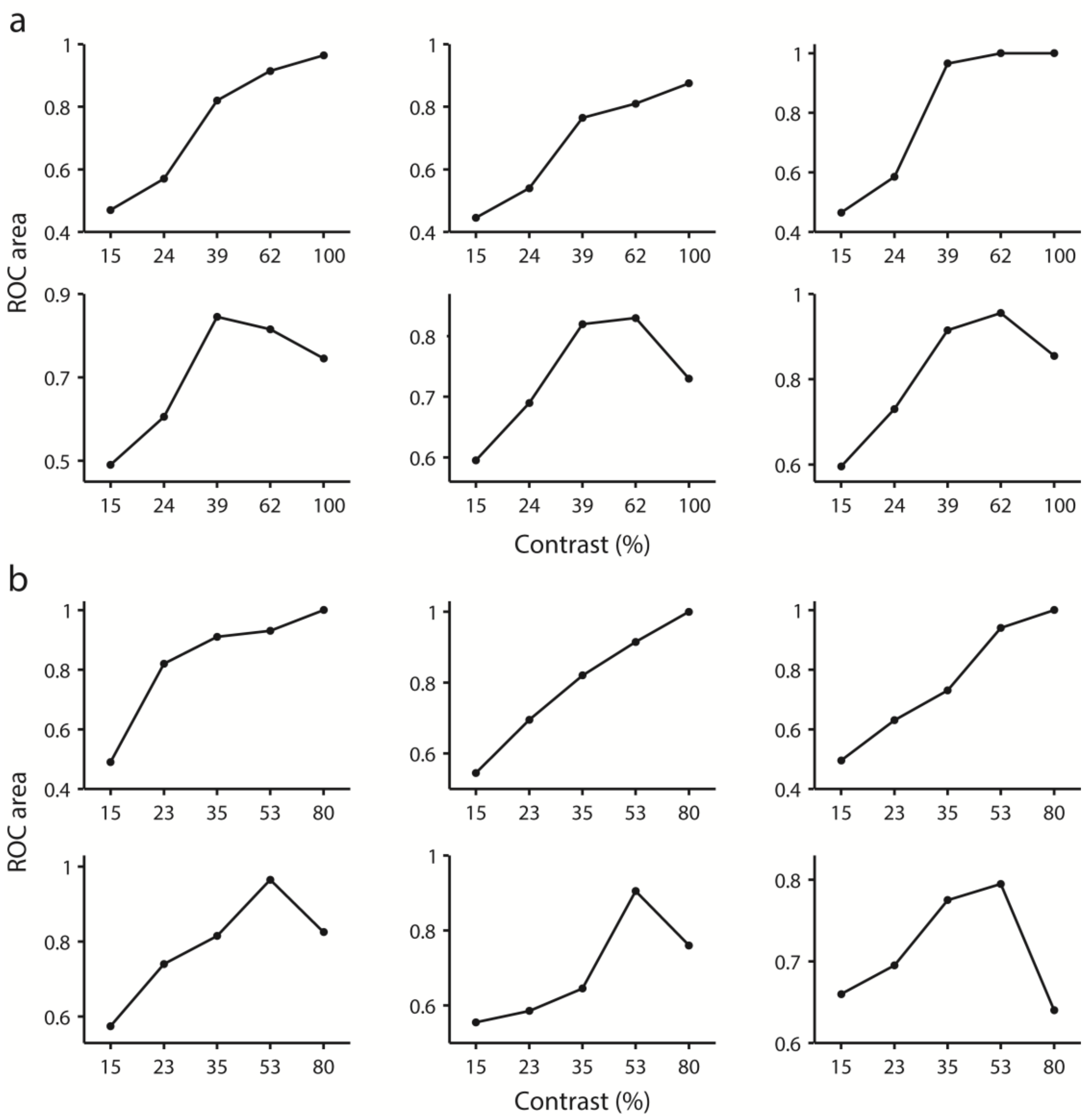

Figure S3. Neurometric functions exhibit a variety of shapes. (a) Neurometric functions for 6 example neurons measured using the contrast range of 15% to 100%. (b) Neurometric functions for 6 example neurons measured using the contrast range of 15% to 80%.

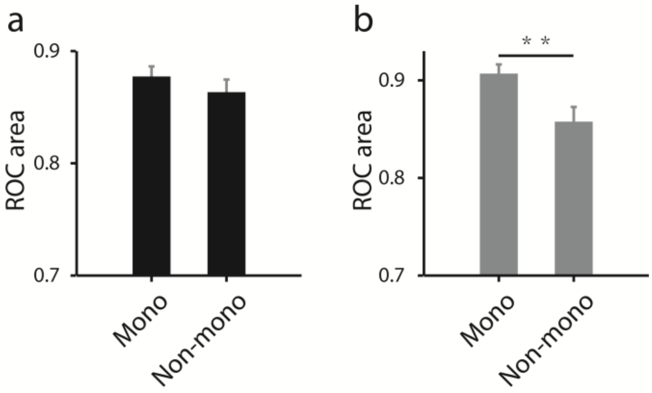

Figure S4. Comparison of the maximum ROC area between cells whose peak ROC areas were at the highest contrast and those cells whose peak ROC areas were at lower contrasts. (a) The responses were measured with contrasts ranging from 15% to 100%. $P = 0.34$, Wilcoxon rank sum test, $n = 135$ for the monotonic cells (cells whose peak ROC areas were at the highest contrast) and $n = 102$ for the non-monotonic cells (cells whose peak ROC areas were not at the highest contrast). (b) The responses were measured with contrasts ranging from 15% to 80%. $P = 0.007$, Wilcoxon rank sum test, $n = 93$ for the monotonic cells and $n = 55$ for the non-monotonic cells. Error bars, s.e.m., **, $P < 0.01$.

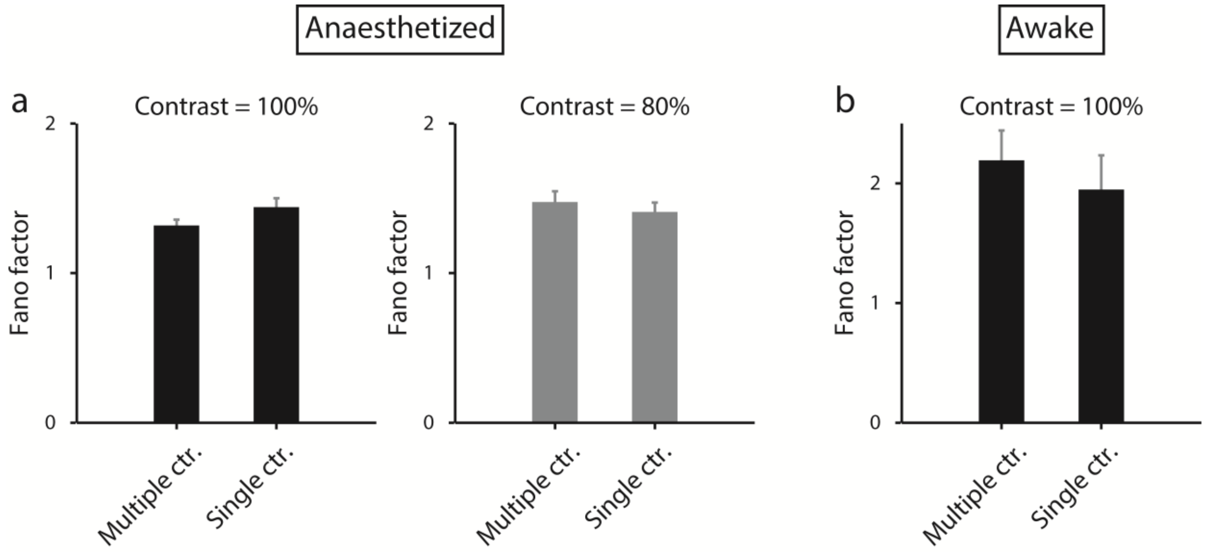

Figure S5. Comparison of the Fano factors between responses in the single and the multiple contrast conditions. Fano factor (FF) was computed using the spike count binned at 667 ms (one cycle of the drifting grating). For each neuron, the FFs for the preferred and the orthogonal orientations were averaged. (a) Left, FF for the responses to 100% contrast in anaesthetized mice. $P = 0.09$, $n = 87$, Wilcoxon signed rank test. Right, FF for the responses to 80% contrast in anaesthetized mice. $P = 0.46$, $n = 92$, Wilcoxon signed rank test. (b) FF for the responses to 100% contrast in awake mice. $P = 0.07$, $n = 17$, Wilcoxon signed rank test. Error bars, s.e.m.

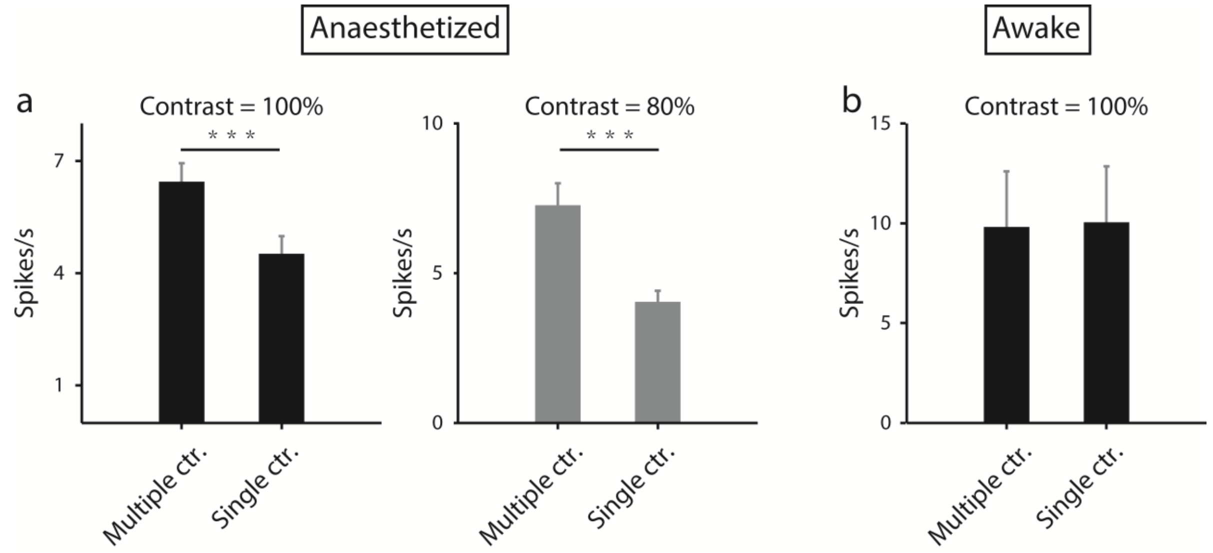

Figure S6. Comparison of the average response to high contrast stimuli between the single and the multiple contrast conditions. (a) Responses in anaesthetized mice. Left, contrast = 100%, $P = 8.4\times10^{-10}$, $n = 87$, Wilcoxon signed rank test. Right, contrast = 80%, $P = 1.2\times10^{-5}$, $n = 92$, Wilcoxon signed rank test. (b) Responses in awake mice. Contrast = 100%, $P = 0.55$, $n = 17$, Wilcoxon signed rank test. Error bars, s.e.m., ***, $P < 0.001$.

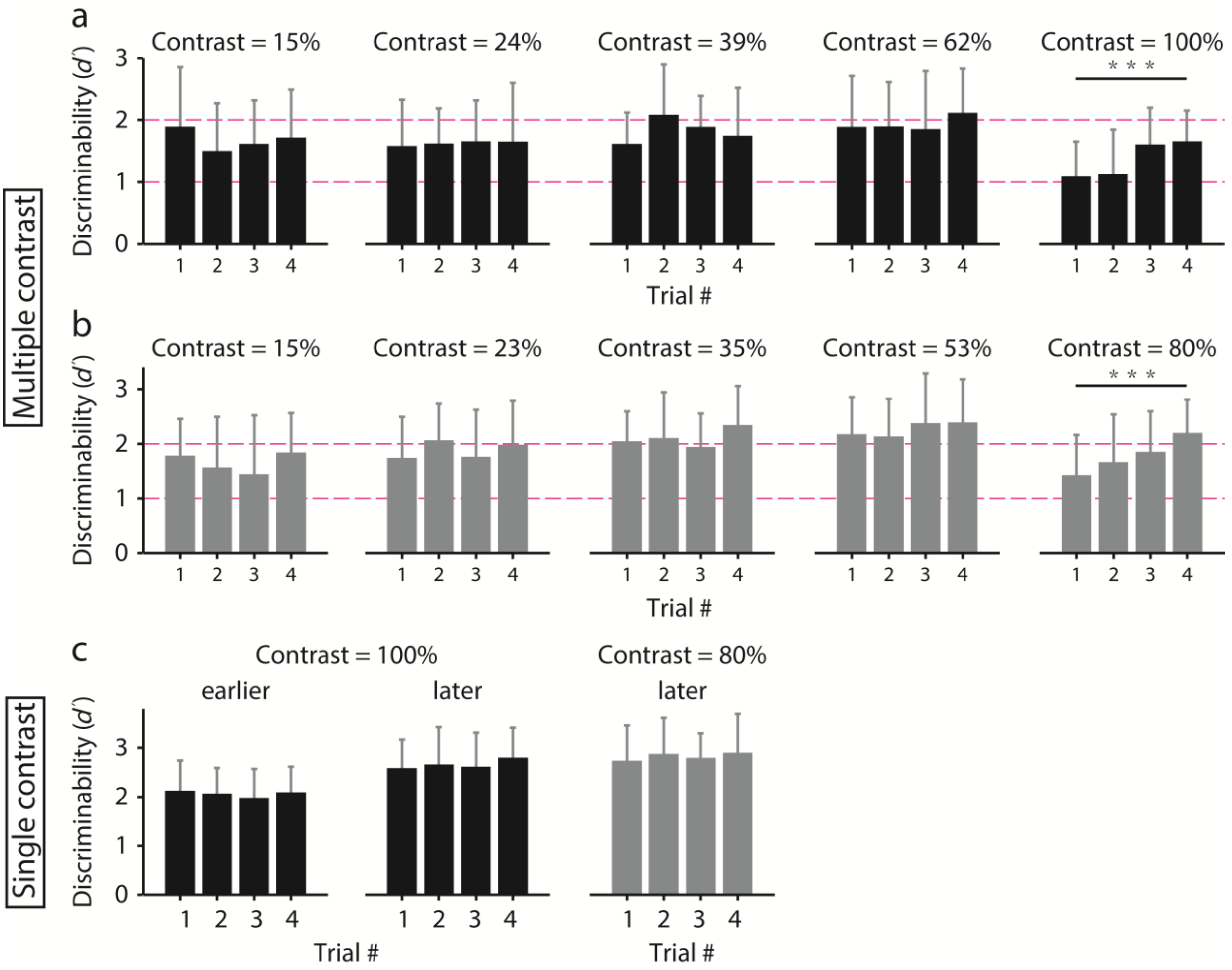

Figure S7. Behavioural performance of the four trials with the same contrast within each block in the multiple contrast condition and that of the four trials within each block in the single contrast condition for the go/no-go task. For all conditions, each block of trials included two targets and two non-targets of the same contrast and the sequence of stimuli within each block were randomized. For the multiple contrast condition, the contrasts in different blocks were also randomized. (a) Multiple contrast condition, contrasts ranging from 15% to 100%. For 100% contrast, the discriminability increased over the four trials (Spearman's rank correlation coefficient r = 1, P = 0.04, n = 14). For the lower contrasts, the discriminability among the four trials were not significantly different (P = 0.63, 0.99, 0.3, and 0.81, respectively, n = 14, ANOVA). (b) Similar as described in (a) except that the contrasts ranged from 15% to 80%. For 80% contrast, the discriminability increased over the four trials (Spearman's rank correlation coefficient r = 1, P = 0.04, n = 14). For the lower contrasts, the discriminability among the four trials were not significantly different (P = 0.58, 0.6, 0.47, and 0.75, respectively, n = 14, ANOVA). (c) Single contrast condition. Left: contrast = 100%, tested before the multiple contrast condition. Middle: contrast = 100%, tested after the multiple contrast condition. Right: contrast = 80%, tested after the multiple contrast condition. For both single contrast conditions, the discriminability among the four trials were not significantly different (P > 0.8, ANOVA). Error bars, s.d., ***, P < 0.001, Wilcoxon signed rank test.
